# Encapsulating metal organic framework into hollow mesoporous carbon sphere as efficient oxygen bifunctional electrocatalyst

**DOI:** 10.1093/nsr/nwz166

**Published:** 2019-11-11

**Authors:** Wanfeng Xiong, Hongfang Li, Hanhui You, Minna Cao, Rong Cao

**Affiliations:** 1 State Key Laboratory of Structural Chemistry, Fujian Institute of Research on the Structure of Matter, Chinese Academy of Sciences, Fuzhou 350002, China; 2 College of Chemistry and Materials Science, Fujian Normal University, Fuzhou 350007, China

**Keywords:** metal organic frameworks, yolk–shell structure, bifunctional electrocatalyst, Zn–air battery

## Abstract

Applying metal organic frameworks (MOFs) in electrochemical systems is a currently emerging field owing to the rich metal nodes and highly specific surface area of MOFs. However, the problems for MOFs that need to be solved urgently are poor electrical conductivity and low ion transport. Here we present a facile *in situ* growth method for the rational synthesis of MOFs@hollow mesoporous carbon spheres (HMCS) yolk–shell-structured hybrid material for the first time. The size of the encapsulated Zeolitic Imidazolate Framework-67 (ZIF-67) is well controlled to 100 nm due to the spatial confinement effect of HMCS, and the electrical conductivity of ZIF-67 is also increased significantly. The ZIF@HMCS-25% hybrid material obtained exhibits a highly efficient oxygen reduction reaction activity with 0.823 V (*vs.* reversible hydrogen electrode) half-wave potential and an even higher kinetic current density (*J*_K_ = 13.8 mA cm^−2^) than commercial Pt/C. ZIF@HMCS-25% also displays excellent oxygen evolution reaction performance and the overpotential of ZIF@HMCS-25% at 10 mA cm^−2^ is 407 mV. In addition, ZIF@HMCS-25% is further employed as an air electrode for a rechargeable Zn–air battery, exhibiting a high power density (120.2 mW cm^−2^ at 171.4 mA cm^−2^) and long-term charge/discharge stability (80 h at 5 mA cm^−2^). This MOFs@HMCS yolk–shell design provides a versatile method for the application of MOFs as electrocatalysts directly.

## INTRODUCTION

With the rapid development of industrial technology, the energy crisis caused by the shortage of fossil energy has been a growing headache. The renewable and green energy source systems such as fuel cell and metal–air batteries are regarded as the reliable alternatives to fossil fuels [1,2]. Oxygen reduction reaction (ORR) and oxygen evolution reaction (OER) are vital semi-reactions in these applications [3–6]. The noble metal catalysts are widely used for both ORR and OER [7]. However, their scarcity, high cost and poor durability strongly impede the large-scale application. Therefore, a rational design of bifunctional non-expensive oxygen electrocatalysts is highly desired.

Metal organic frameworks (MOFs), a new class of material with special chemical and physical properties, have attracted tremendous attention in recent years for their versatile potential applications in gas storage and separation [8], catalysis [9], sensors [10], drug delivery [11] and so on. Recently, applying MOFs in electrochemical reactions has been an emerging research field because the high surface area of MOFs can maximize active site density, and the special chemical structures of MOFs provide a tailored microenvironment for controllable reaction within the pores [12]. However, it is rarely reported to use MOFs directly in the electrocatalysis field due to their low ion transport and unfavourable electrical conductivity [13]. To address these issues, great efforts have been focused to develop efficient MOF-derived electrode materials such as metal oxides [14], metallic nanoparticles [15,16] and porous carbon compounds [17] through pyrolysis. This method can effectively improve the conductivity of materials and create active sites. However, the metal nodes are prone to agglomeration, and the ordered porosity will be destroyed during thermal treatments, which will limit catalytic activity more or less. Another method for enhancing the conductivity of pristine MOFs is to combine MOFs with various conductive carbon materials, including porous carbon [18], carbon nanotubes [19] and graphene [20]. Nevertheless, non-uniform dispersion of the second carbon phase cannot increase electrical conductivity effectively. Moreover, complete coverage of MOFs with carbon materials will also partly reduce the active sites of MOFs. Therefore, how to solve the low ion transport and the poor electrical conductivity of MOFs simultaneously is still the bottleneck of MOFs electrocatalysis.

Encapsulating nanoparticles into a hollow mesoporous carbon sphere (HMCS) is a classical design. This design is helpful to stabilize catalytic active sites, increase electrical conductivity and reduce mass transport lengths [21]. The yolk–shell structure design such as metallic nanoparticles@carbon, metal oxide@carbon has been widely used in lithium batteries, catalysis and other fields [22–24]. However, the design of MOFs@HMCS yolk–shell-structured hybrid material has not been reported yet. Therefore, it is believed that the elaborate combination of MOFs with HMCS to construct a yolk–shell structure hybrid material will effectively overcome the previously mentioned shortcoming of MOFs materials in the electrocatalysis field.

It is well known that ZIF-67 is a zeolitic imidazolate framework (ZIF) constructed with Co^2+^ ions and 2-methylimidazole (2-MI). Due to the inherent high activity structural motif Co-N_4_, ZIF-67 has been proved to be a potential catalyst in both ORR and OER [25,26]. However, the huge size and low conductivity of ZIF-67 weaken its catalytic activity. Here, a yolk–shell-structured ZIF-67@HMCS hybrid material is innovatively designed by using ZIF-67 as core and HMCS as shell. The particle size of ZIF-67 is well controlled by utilizing the spatial confinement effect from HMCS, which shortens the diffusion paths and enhances the ion transportation. Encapsulating ZIF-67 in HMCS also increases its conductivity prominently. Moreover, the typical hierarchical pore structures of HMCS guarantee the diffusion of reactive species to the exposed active sites of ZIF-67 quickly and efficiently, and thus improve the electrochemical activity. The electrochemical catalytic performances of such yolk–shell-structured ZIF@HMCS-25% has been intensively studied and ZIF@HMCS-25% exhibits superior bifunctional electrocatalytic activity towards both ORR and OER. What is more, the assembled Zn–air battery by using ZIF@HMCS-25% as air-cathode also presents impressive performance and long-term stability.

## RESULTS AND DISCUSSION

**Scheme 1.**
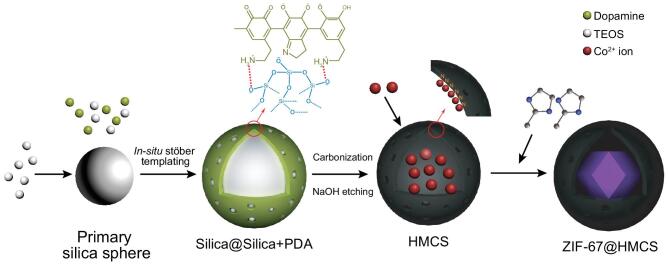
Schematic illustration of synthetic procedure for ZIF@HMCS.

The synthesis process of ZIF@HMCS hybrid materials is illustrated in Scheme 1. Firstly, SiO_2_ spheres and dopamine were used as template and carbon source, respectively, to synthesize HMCS according to the modified method from Owen Noonan [27]. As shown in Fig. S1a in the online supplementary material, HMCS exhibited a uniform spherical morphology with a loose surface. The carbon shell was extremely thin, ∼10 nm. The cavity was huge, up to 246 nm (Fig. S1e), which provided enough space for the growth of ZIF-67. Elemental analysis (Table S1 in the online supplementary material) exhibited a high N content of 6.4% doping in HMCS. The introduction of N heteroatoms definitely caused the distortion of carbon rings such as the formation of pyridinic-N or pyrrolic-N, and therefore resulted in large numbers of defects in the carbon matrix. The existence of these defects was further confirmed by a Raman spectrum. As shown in Fig. S2a in the online supplementary material, *I*_D_ was stronger than *I*_G_ and the ratio of *I*_D_/*I*_G_ was 1.067, which verified the distortion of carbon rings in HMCS. In order to verify the anchor effect of N sites, the parallel sample Co-HMCS was prepared. The HMCS was treated with Co^2+^ ion precursor following the same process as ZIF@HMCS-25% without the addition of 2-MI. The result of inductively coupled plasma atomic emission spectrometry (ICP) showed a Co content of 1.16% in Co-HMCS. The interaction between Co^2+^ ions with the doping N in HMCS was further confirmed by deconvoluted X-ray photoelectron spectroscopy (XPS) N 1s spectra (Fig. S2b). Therefore, abundant N-doped sites in HMCS were ready to capture Co^2+^ and induced ZIF-67 to grow in the cavity of HMCS. Further regulating the quantity of HMCS, Co(NO_3_)_2_·6H_2_O and 2-MI, a series of ZIF@HMCS-m (m: weight ratio of HMCS to Co(NO_3_)_2_·6H_2_O) hybrid materials were obtained.

**Figure 1.**
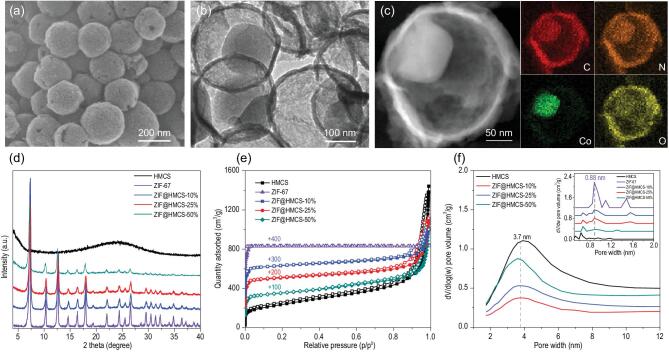
(a) SEM image of ZIF@HMCS-25%. (b) TEM image of ZIF@HMCS-25%. (c) HAADF-STEM images and EDS mappings of ZIF@HMCS-25%. (d) PXRD patterns of HMCS, ZIF-67 and ZIF@HMCS-m hybrid materials. (e) N_2_ adsorption–desorption isotherms of HMCS, ZIF-67 and ZIF@HMCS-m hybrid materials. (f) BJH pore size distributions of HMCS and ZIF@HMCS-m (inset: NLDFT pore size distributions of HMCS, ZIF-67 and ZIF@HMCS-m).

The powder X-ray diffraction (PXRD) patterns of all the samples are presented in Fig. 1d. All of the diffraction peaks could be exclusively assigned to ZIF-67. No obvious diffraction peaks corresponding to amorphous carbon were found in the PXRD patterns of ZIF@HMCS-m samples, which might be shielded by the sharp ZIF-67 diffraction peaks. Increasing the content of HMCS in the hybrid materials, the peak intensity of ZIF-67 was gradually weakened and the Co and N content decreased correspondingly (Table S1), which was consistent with the feed ratio.

The morphologies of hybrid materials could be observed directly from transmission electron microscopy (TEM) and scanning electron microscopy (SEM) images. As illustrated in Fig. 1a, ZIF@HMCS-25% maintained the original spherical morphology of HMCS. No obvious dodecahedral-structured ZIF-67 particles could be found outside the spheres. TEM and high-angle annular dark-field scanning transmission electron microscopy (HAADF-STEM) (Fig. 1b and c) images showed that the particles of ZIF-67 ∼100 nm grew close to the inner wall of HMCS. This phenomenon could be attributed to the doping N sites in HMCS acting as an anchor for Co^2+^ precursor and therefore provided the initial growth point for ZIF-67. The STEM and energy-dispersive X-ray spectroscope (EDS) mapping images (Fig. 1c) showed that Co element signals were quite matched with the ZIF-67 particle, and N and C elements exhibited an obvious signal enhancement in this area. The weak O signals could be attributed to the adsorption of O_2_ on the surface of ZIF-67 [28]. This evidence further confirmed the successful synthesis of a yolk–shell-structured ZIF@HMCS hybrid material.

For comparison, pure ZIF-67, ZIF@HMCS-10% and ZIF@HMCS-50% were also characterized by SEM and TEM. Pure ZIF-67 was readily grown into huge particles ∼1 μm (Fig. S1b and f). When 10% HMCS was introduced, the size of ZIF-67 decreased significantly to ∼200 nm. However, low content of HMCS could not provide enough confined space for the growth of ZIF-67 and there were still several ZIF-67 particles existing outside the HMCS (Fig. S1c and g). Further increasing HMCS content to 50%, only a few ZIF-67 particles could be observed due to the lack of Co^2+^ precursor (Fig. S1d and h). Therefore, the mass ratio between HMCS and Co(NO_3_)_2_·6H_2_O was quite important for the successful synthesis of yolk–shell-structured ZIF@HMCS hybrid materials and the optimized feed ratio was 25%.

It is worth noting that a suitable HMCS substrate played a decisive role in the construction of the yolk–shell structure. When HMCS-1, which had a thicker shell (∼30 nm) and a dense surface (Fig. S3a and c) was used as a carbon substrate, the Co^2+^ and 2-MI were hardly infiltrated into the cavity. All ZIF-67 particles grew outside HMCS-1 (Fig. S3b and d) and there were no ZIF-67 particles synthesized in the cavity. It is well known that a high reaction temperature will result in a larger ZIF-67 particle size. Raising the reaction temperature to 150°C, the obtained ZIF-67@HMCS hybrid material preserved the yolk–shell structure and the particle size of ZIF-67 had no obvious increase (Fig. S4a in the online supplementary material). Therefore, the growth of ZIF-67 was fully restricted in the cavity of HMCS. The confinement effect of HMCS could be further verified by a ZIF@BHMCS (broken hollow mesoporous carbon spheres)-25% sample, whose hollow sphere structure was completely destroyed during long-duration ultrasonic treatment. As can be seen in Fig. S4b, only huge ZIF-67 particles existed outside broken HMCS in ZIF@BHMCS-25%. Therefore, the optimized feed ratio and suitable HMCS substrate, as well as the confinement growth of ZIF-67, guaranteed the successful synthesis of a yolk–shell-structured ZIF@HMCS hybrid material.

N_2_ adsorption–desorption isotherms were used to characterize the pore structures of the hybrid materials. As illustrated in Fig. 1e, all samples exhibited a hierarchical pore structure characteristic adsorption. The hysteresis loops at higher relative pressure were attributed to macropores adsorption and displayed a significant decrease in hybrid materials, which certified the occupation cavity of HMCS by ZIF-67 particles. The pore size distribution of ZIF@HMCS-m hybrid materials all exhibited bimodal pore sizes centered at 3.7 and 0.88 nm, respectively. The larger mesopores were derived from the carbon shell and the smaller micropores were originated from the ZIF-67 (Fig. 1f). The specific surface areas of ZIF@HMCS-m hybrid materials were all high, up to 800 cm^2^ g^−1^ (Table S1). What is more, micropore and mesopore volumes of hybrid materials were readily regulated by adjusting the content of HMCS. Such hierarchical pore structures were important to promote the accessibility of active sites and mass transfer during the ORR and OER catalytic process [29].

**Figure 2.**
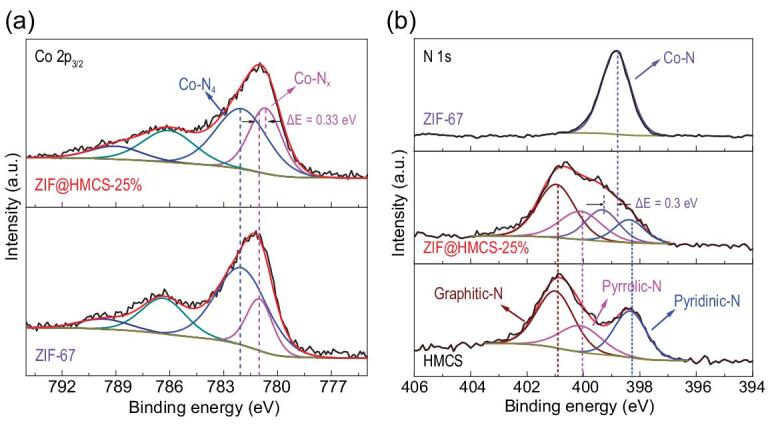
(a) Deconvoluted XPS Co 2p_3/2_ spectra of ZIF-67 and ZIF@HMCS-25%. (b) Deconvoluted XPS N 1s spectra of HMCS, ZIF-67 and ZIF@HMCS-25%.

The chemical coordination environment of ZIF-67 and ZIF@HMCS-25% was intensively studied by XPS. It was obvious that the Co 2p_3/2_ peak of ZIF@HMCS-25% showed a down-shift, and its half-width was significantly increased (Fig. 2a), indicating some changes in the coordination environment of Co^2+^ ions [30]. Deconvoluted Co 2p_3/2_ spectra both exhibited four peaks for ZIF-67 and ZIF@HMCS-25%. The peaks at 781.1 eV and 782.7 eV in ZIF-67 represented Co-N*_x_* (*x* < 4) and the Co-N_4_ coordination environment, respectively, and the other two peaks were regarded as satellite peaks [31]. The Co-N*_x_* coordination was mainly originated from the unsaturated coordination of Co^2+^ ions on the outer surface of ZIF-67 [25]. Different from ZIF-67, Co-N*_x_* content in ZIF@HMCS-25% significantly increased from 25.4% to 40.6%, which was attributed to a decrease in size of ZIF-67. Smaller ZIF-67 particles have larger external surface area and made more Co^2+^ ions exposed to the outer layer, and therefore result in the increase of unsaturated coordination (Co-N*_x_*). Meanwhile, the Co-N*_x_* peak in ZIF@HMCS-25% showed an obvious down-shift (0.33 eV) compared with ZIF-67, which proved that surface N coordinate sites changed partially from 2-MI to pyridinic-N or pyrrolic-N in HMCS. In addition, the XPS spectrum of N 1s in the ZIF@HMCS-25% sample (Fig. 2b) could be fitted into four peaks that correspond to pyridinic-N, pyrrolic-N, graphitic-N and Co-coordinate N (Co-N) functional groups, respectively. Obviously, the Co-N peak also showed a significant up-shift of ∼0.3 eV, which was consistent with the results of Co 2p_3/2_ spectra (Fig. 2b). The XPS results confirmed the interaction between ZIF-67 and HMCS. Tailoring the surface and electronic structure of Co^2+^ with the doping N atoms in HMCS would improve the interactions between metal active sites and substrate and thus enhance their catalytic activity [32–34].

**Figure 3.**
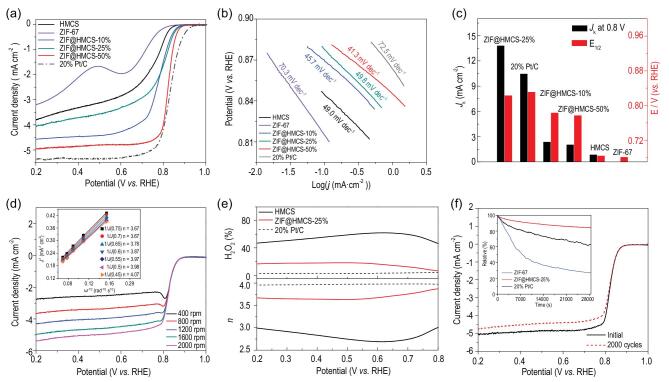
(a) LSV curves toward ORR and (b) ORR Tafel plots of various samples. (c) Comparison of half-wave potential and *J*_K_ for different catalysts. (d) LSV curves toward ORR of ZIF@HMCS-25% at different rotation rates. (e) H_2_O_2_ (%) yield (top) and electron transfer number (*n*, bottom). (f) ADTs of ZIF@HMCS-25% before and after 2000 potential cycles (inset was chronoamperometric responses of ZIF-67, ZIF@HMCS-25% and 20 wt% Pt/C at 0.6 V *vs.* RHE).

The linear scan voltammogram (LSV) curves were used to evaluate ORR activities for ZIF-67, HMCS and ZIF@HMCS-m samples. As shown in Fig. 3a, ZIF-67 and HMCS showed poor ORR activities. Nevertheless, the catalytic activities of ZIF@HMCS-m hybrid materials significantly improved. Among all samples, ZIF@HMCS-25% exhibited the best activity with the most positive half-wave potential of 0.823 V (*vs.* RHE), which was close to the commercial 20 wt% Pt/C (0.831 V) and exceeded most of the other MOF-based catalysts (Table S2 in the online supplementary material). The ZIF@HMCS-25% also exhibited a large diffusion-limited current density, which was competitive with that of the Pt/C catalyst. The Tafel slope obtained by the *log|i|-E* relationship from the polarized region indicated the kinetic properties of samples (Equations (5–7) in Methods section). As shown in Fig. 3b, ZIF@HMCS-25% revealed a lowest Tafel slope of 41.9 mV dec^−1^, which was much lower than that of the Pt/C (72.5 mV dec^−1^), suggesting its superior catalytic efficiency for the ORR. Comparing with ZIF-67 (70.3 mV dec^−1^), the reducing Tafel slopes of ZIF@HMCS-m probably originated from the hierarchical pore structure of HMCS. The hierarchical pore structure promoted the diffusion of electrolytes and therefore increased the kinetic efficiency of hybrid materials [35]. The kinetic current density (*J*_K_) at 0.8 V (*vs.* RHE) obtained from the Koutecky–Levich (K-L) equation reached up to 13.8 mA cm^−2^, which was higher that of the commercial Pt/C (10.4 mA cm^−2^), further suggesting the superior ORR efficiency of ZIF@HMCS-25% (Fig. 3c). The K-L slopes of ZIF@HMCS-25% obtained from LSV curves of various rotating speeds (Fig. 3d) presented nearly parallel fitting lines, demonstrating O_2_ first-order reaction kinetics and a potential-independent electron transfer rate. The electron transfer number obtained at different potentials suggested a four-electron pathway for ORR. Rotating ring-disk electrode (RRDE) measurement (Fig. 3e) was also performed to investigate the electron transfer number (*n*) and H_2_O_2_ (%) yield. The ZIF@HMCS-25% exhibited an electron transfer number ∼3.7 and H_2_O_2_ (%) yield ∼15%, which was consistent with the results of K-L slopes.

The durability of ZIF@HMCS-25% was evaluated by accelerated durability tests (ADTs) (Fig. 3f) and chronoamperometry (inset of Fig. 3f). The ADTs result of ZIF@HMCS-25% demonstrated that half-wave potential was only 4 mV declined after 2000 times cyclic voltammetry (CV) scans and the current density decreased slightly. The chronoamperometric responses showed that ZIF@HMCS-25% remained 84.5% of current density after a 28 000 s durability test. However, only 26.5% for the ZIF-67 and 62.5% for the Pt/C could be retained at the same condition, suggesting good stability of ZIF@HMCS-25% in alkaline medium. The structural stability of ZIF@HMCS-25% was characterized by PXRD and TEM. The PXRD pattern (Fig. S5a in the online supplementary material) exhibited three strongest peaks attributed to ZIF-67 after the stability test, TEM images (Fig. S5b) showed that the morphology of the ZIF-67 core and HMCS shell were still maintained, which indicated the excellent structural stability of ZIF@HMCS-25%.

For comparison, the ORR activity of three contrasting samples including Co-HMCS, ZIF/HMCS-25% and ZIF@BHMCS-25% were also tested in the same condition. Co-HMCS displayed a significant performance improvement compared with HMCS, but still could not catch up with ZIF@HMCS-25% (Fig. S6 in the online supplementary material). This evidence further indicated the role of the ZIF-67 core in boosting ORR performance. As can be seen from Fig. S6, the half-wave potential and diffusion limiting current (*J*_L_) of ZIF/HMCS-25% and ZIF@BHMCS-25% both decreased obviously. Therefore, it was evident that the unique yolk–shell structure of ZIF@HMCS was a benefit for the ORR reaction. The hierarchical porous structure of HMCS facilitated quick diffusion of reactive species to active sites effectively. The size decrease of ZIF-67 shortened diffusion paths and enhanced ion transportation.

It was reported previously that N and Co species were catalytically active for ORR. ZIF-67 had abundant Co-N_4_ sites in the framework and it was an intrinsic catalyst for ORR. The hybrid composite ZIF@HMCS retained Co-N_4_ sites as much as possible. Moreover, owing to the interaction between ZIF-67 and HMCS, the Co-N*_x_* sites with higher ORR activity were increased and also played the catalytic role in the ORR process. On the other hand, N-doping HMCS also had ORR activities (Fig. 3a) due to the existence of abundant pyridinic-N [36,37]. Therefore, it was confirmed that the synergistic effect between Co-N*_x_* (*x* ≤ 4) and doping N atoms in ZIF@HMCS hybrid material contributed to the high ORR activity.

**Figure 4.**
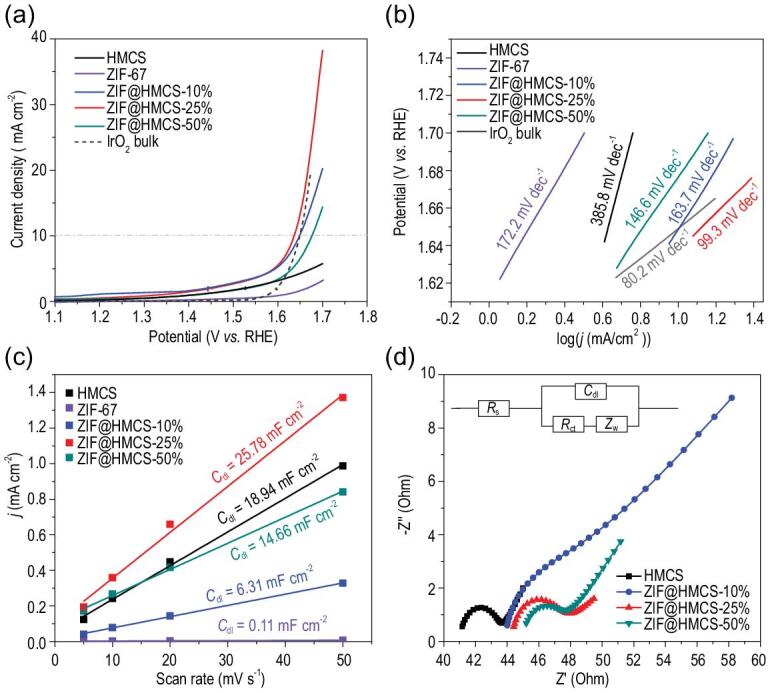
(a) LSV curves toward OER and (b) OER Tafel plots of various samples. (c) Double-layer capacitance (*C*_dl_) obtained by a different scan rate for different samples. (d) High frequency range EIS after fitting of various samples (inset: the corresponding equivalent circuit diagram).

The unique yolk-shell structure of ZIF@HMCS could boost electrocatalysis, which was also verified by the electrocatalytic OER. It was clear that the current density of pure HMCS and ZIF-67 material could not reach 10 mA cm^−2^ even at a very high potential of 1.7 V (*vs.* RHE) and their Tafel slope was higher than the hybrid materials **(**Fig. 4a and b**)**. Therefore, pure HMCS and ZIF-67 exhibited poor OER catalytical activity. Wrapping ZIF-67 with HMCS improved the catalytic activity of ZIF@HMCS-m hybrid materials. The ZIF@HMCS-25% also exhibited the best OER activity among all the hybrid materials. It showed a lower overpotential of 407 mV than commercial IrO_2_ bulk of 420 mV at 10 mA cm^−2^ (Fig. 4a). The Tafel slope of ZIF@HMCS-25% was 99.3 mV dec^−1^ (Fig. 4b), it was close to IrO_2_ bulk (80.3 mV dec^−1^) and other MOF-based catalysts (Table S2). The OER durability of ZIF@HMCS-25% was also evaluated. As shown in Fig. S7a in the online supplementary material, ZIF@HMCS-25% displayed a better performance than IrO_2_ bulk in the chronopotentiometry durability test. After the durability test, the structure of ZIF@HMCS-25% was characterized by PXRD, XPS and TEM. As shown in Fig. S7b, the diffraction peaks assigned to ZIF-67 disappeared after the OER durability test. By analyzing the Co 2p_3/2_ XPS spectra of the tested ZIF@HMCS-25% (Fig. S7c), the Co-N*_x_* and Co-N_4_ sites significantly decreased and the conversely Co-O/CoC*_y_*N*_x_* sites appeared [38]. The TEM image also showed that the ZIF-67 particles were invisible (Fig. S7d). According to the characterization results, it can be supposed that ZIF-67 was converted into an amorphous Co(OH)_2_ and loaded on the HMCS during the OER process. A similar phenomenon has been observed in previous work and this (oxy)hydroxide phase has also been proved to be a highly active site for OER [39].

To further explore the reason for the high catalytic activity of ZIF@HMCS-25%, electrochemical surface areas (ECSAs) and electrochemical impedance spectroscopy (EIS) were used to evaluate the numbers of active sites and the charge transport kinetics, respectively. The double-layer capacitance (*C*_dl_) obtained from cyclic voltammograms at different scan rates (Fig. S8 in the online supplementary material) was used to evaluate the ECSAs. As seen in Fig. 4c, ZIF@HMCS-25% had a good *C*_dl_ performance of 25.79 mF cm^−2^, which indicated more active sites could be exposed and used in the electrochemical catalysis. The promotion of the ECSA could be attributed to the size controlling of ZIF-67, which exposed more active site efficiently. The EIS results showed that ZIF@HMCS-m hybrid materials all had a smaller semicircle trend in the low frequency range indicating their good diffusion property [40], which was likely due to their hierarchical pore structures (Fig. S9 in the online supplementary material). The high frequency range of EIS (Fig. 4d) displayed charge transfer ability for different samples. In order to obtain accurate resistance information, EIS in the high frequency region (100 kHz to 100 Hz) was fitted with an equivalent circuit diagram (inset of Fig. 4d**)**. The results illustrated that charge transfer resistance (*R*_ct_) of ZIF-67 was high to 33.87 Ω. After introduction of HMCS, the *R*_ct_ of ZIF@HMCS-25% decreased dramatically to 3.58 Ω due to the high conductivity of HMCS (Table S3 in the online supplementary material). EIS measurement further confirmed that the yolk–shell structure played a vital role in the improvement of conductivity. In conclusion, ZIF@HMCS-25% had the largest active sites and good conductivity, which ensured its high electrocatalytic activity.

**Figure 5.**
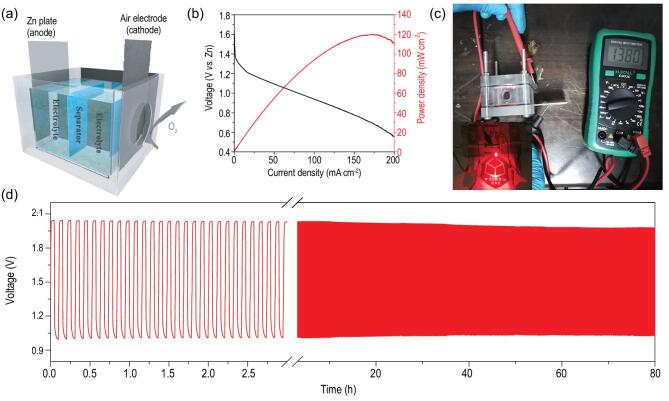
(a) Illustration of assembled Zn–air battery. (b) Polarization curves and the corresponding power density curves of Zn–air battery (catalyzed by ZIF@HMCS-25%). (c) The open-circuit voltage of the Zn–air battery (1.38 V) and a red LED (∼2 V) powered by two primary Zn–air batteries in series. (d) Discharge/charge cycling curves of Zn–air battery using ZIF@HMCS-25% as air electrodes at a current density of 5 mA cm^−2^ (6 min for each cycle).

To evaluate the actual application of ZIF@HMCS-25%, a polished zinc plate and a carbon paper with ZIF@HMCS-25% catalyst loaded were assembled to construct a home-made Zn–air battery **(**Fig. 5a**)**. As shown in Fig. 5b, the primary Zn–air battery exhibited an open-circuit potential of ∼1.38 V and a high peak power density of 120.2 mW cm^−2^ at 171.4 mA cm^−2^. Assembling two batteries in series could light a red light-emitting diode (LED) in the practical application (Fig. 5c). In addition, the efficiency and long-term rechargeability of ZIF@HMCS-25% was evaluated by galvanostatic charge/discharge tests. As shown in Fig. 5d, the initial charge/discharge potentials of the primary Zn–air battery were 2.04 V and 1.00 V with a small voltage gap of 1.04 V at a constant current density of 5 mA cm^−2^. After 80 h cycling (6 min per cycle), almost invariable charge and discharge potentials (1.98 and 1.03 V with a smaller voltage gap of 0.95 V) could be observed, demonstrating its excellent activity and stability.

Based on the discussions above, the excellent electrocatalytic activity of ZIF@HMCS-25% toward both ORR and OER could be attributed to the following characteristic: (1) abundant coordination unsaturated Co-N*_x_* (*x* ≤ 4) species in ZIF-67 served as highly active sites; (2) the hierarchical porous structure and the small particle size of ZIF-67 facilitated the diffusion of reactive species to the exposed active sites quickly and efficiently; (3) the high conductivity of HMCS enhanced the electron transfer capacity; (4) the synergistic effect between ZIF-67 and HMCS contributed to the enhanced elctrochemical catalysis activity; (5) the HMCS shell provided a protection for inner ZIF-67 and led to excellent durability.

## CONCLUSION

In summary, we elaborately designed a yolk–shell-structured ZIF-67@HMCS hybrid material by using ZIF-67 as core and HMCS as shell. This design implemented the encapsulation of MOFs into HMCS successfully for the first time. The HMCS provided a confined space for the growth of ZIF-67 and the particle size of ZIF-67 could be reduced to ∼100 nm. This ingenious yolk–shell design provided abundant coordinatively unsaturated Co-N*_x_* sites, a hierarchically porous structure and significantly enhanced conductivity. Therefore, ZIF@HMCS-25% hybrid material exhibited superior electrocatalytic activity towards both ORR and OER. Furthermore, ZIF@HMCS-25% showed outstanding performance and stability in a Zn–air battery. This bifunctional yolk–shell-structured hybrid material could be a promising candidate as an electrocatalyst in fuel cells and electrolysers for renewable energy applications. This work also paves a new way toward designing stable MOFs used directly as high efficiency electrochemical catalysts in promising energy storage devices to meet the growing demand of stable energy supply.

## METHODS

### Materials

All chemicals and solvents were used directly without further purification. Ammonium hydroxide (NH_4_OH, AR, 25–28 wt%), ethanol (AR), sodium hydroxide (NaOH, AR) were acquired from Sinopharm Chemical Reagent Co. Ltd (China). Tetraethoxysilane (TEOS, 99%), 3-hydroxytyramine hydrochloride (dopamine, 99%), cobalt nitrate hexahydrate (Co(NO_3_)_2_·6H_2_O, 99%), 2-methylimidazole (2-MI, 98%) were purchased from Adamas Reagent, Ltd.

### Preparation of the HMCS

The HMCS was prepared using a modified method of Owen Noonan [27]. In a typical preparation, 6 mL NH_4_OH was added to a mixed solution of 140 mL ethanol and 20 mL H_2_O under stirring. Then, 5.6 mL TEOS was added and reacted under magnetic stirring for 30 min. Following this, 320 mL of an aqueous solution of dopamine (2.5 mg mL^−1^) was slowly injected into the above solution. The mixed solution was further stirred for 12 h to yield a brownish-black solid. The product was centrifuged and washed several times by water and ethanol and dried. Next, the solid product was calcinated at 800°C for 5 h under a nitrogen atmosphere. Finally, the SiO_2_@carbon pellets obtained were etched by 1 M NaOH at 70°C for 24 h to obtain the HMCS. HMCS-1 was obtained following the same procedure but just changing the dopamine aqueous solution from 2.5 to 5.0 mg mL^−1^.

### Preparation of ZIF-67@HMCS

The as-prepared HMCS (10 mg) was dispersed in various volumes (10 mL, 4 mL and 2 mL) of Co(NO_3_)_2_ methanol solution (10 mg mL^−1^). After ultrasonic dispersion for 30 min, the mixture solution was transferred to a shaker and was shaken overnight. Then different amounts (10 mL, 4 mL and 2 mL) of 2-MI methanol solution (13.12 mg mL^−1^) were added. The mixture was stirred vigorously, and then transferred to a 5°C bath for 24 h. Finally, the black powder was obtained by centrifugation and washed several times until the supernatant was colorless. The obtained hybrid materials were denoted as ZIF@HMCS-m, where m is the weight ratio of HMCS to Co(NO_3_)_2_·6H_2_O. For optimization the synthetic condition, ZIF@HMCS-1-25% was prepared by following the same process but selecting HMCS-1 as substrate. The ZIF@BHMCS-25% sample was synthesized by prolonging the ultrasonic treatment time from 30 min to 2 h, which resulted in the broken HMCS shell and impeded the formation of a yolk–shell structure. In addition, the effect of synthetic temperature on the structure of ZIF@HMCS-25% was also studied extensively by changing the temperature from 5°C to 150°C. As reference, pure ZIF-67 was synthesized through a similar procedure without the addition of HMCS. The physical mixture of ZIF-67 and HMCS was also prepared and labeled as ZIF/HMCS-25%.

### Preparation of Co-HMCS

Similar to the preparation of ZIF@HMCS-25%, the as-prepared HMCS (10 mg) was dispersed in 4 mL Co(NO_3_)_2_ methanol solution (10 mg mL^−1^). After ultrasonic dispersion for 30 min, the mixture solution was transferred to the shaker and shaken overnight and then transferred to a 5°C bath for 24 h. Finally, the black powder was obtained by centrifugation and washed several times until the supernatant was colorless. The obtained materials were denoted as Co-HMCS.

### Characterization

TEM and HAADF-STEM images were acquired with a JEOL-2010 FEI Tecnai G20 field-emission microscope (JEOL, Tokyo, Japan) operated at 200 kV. SEM images were obtained by field emission scanning electron microscopy (JSM6700). ICP was performed on an Ultima2. PXRD patterns were recorded on a Miniflex 600 X-ray diffractometer under Cu Kα radiation (*λ* = 1.5406). The Raman spectra were performed on a Labram HR800 Evolution over a range of 300–3500 cm^−1^. The N_2_ adsorption–desorption isotherms of the samples were collected using a Micromeritics ASAP 2460 instrument. The micropore size distributions of the samples were calculated via a non-local density functional theory (NLDFT) method and the mesopore size distributions were calculated by the Barrett–Joyner–Halenda (BJH) adsorption isotherms. The specific surface areas were measured using the Brunauer–Emmett–Teller (BET) model. Elemental analyses were measured by a vario EL cube (Elementar, Germany). XPS was performed on an ESCALAB 250Xi X-ray photoelectron spectrometer (Thermo Fisher) using monochromatized Al Kα radiation (15 kV, 10 mA).

### Electrochemical experiments

All the electrochemical experiments were conducted in 0.1 M KOH (pH = 13) on an IM6ex workstation (Zahner, Germany) by using a rotating disk electrode (RDE) or a RRDE (PINE, USA). A three-electrode system by using Pt foil and Ag/AgCl electrode as the counter electrode and the reference electrode was applied in electrochemical measurement respectively. The conversion between measured potentials and RHE scale was by the following equation: *E*_RHE_ = *E*_vs. Ag/__AgCl_ + 0.0592pH + 0.197. Before all the electrochemical measurements, electrolyte solutions were purged with O_2_ (or N_2_) for at least 30 min to make the solution reach O_2_-saturated (or N_2_-saturated). The as-prepared catalyst (2.5 mg) was mixed with 1 mL isopropanol and 40 μL Nafion solution (5 wt%, Adamas) and ultrasonic treatment for 30 min to yield a homogeneous ink. Then, 20 μL of the resulting ink was dropped onto the surface of the glassy carbon disk (0.197 cm^2^) and dried at room temperature. The total loading of the catalyst was about 0.253 mg cm^−2^.

### Measurement for the ORR

The catalyst was firstly treated in a N_2_-saturated 0.1 M KOH solution by CV scans at 100 mV s^−1^ for about 10–20 cycles until the signals were stable. Next the background current was tested by LSV scan with a sweep rate of 10 mV s^−1^. After that, the working electrode was transferred into an O_2_-saturated 0.1 M KOH solution by CV scans at 100 mV s^−1^ for about 10 cycles. Then its ORR current was tested by an LSV scan with a sweep rate of 10 mV s^−1^ from 0.2 to −0.8 V (*vs.* Ag/AgCl) after iR-compensation. The final resulting ORR catalytic activity curves were background subtracted. For the stability measurement, the test environment was the same as the ORR reaction test (O_2_-saturated) at a rotation rate of 1600 rpm. The chronoamperometric test lasted 25 000 s at a constant potential of 0.6 V (*vs*. RHE). The ADTs were evaluated by the change in LSV curves before and after 2000 cycles of CV scans between 0.6 and 1.0 V (*vs.* RHE) at a sweep rate of 50 mV s^−1^.

The K-L equation was used to obtain the electron transfer number (*n*) and kinetic current density (*J*_K_):

(1) \begin{equation*}\frac{1}{J} = \frac{1}{{{J_{\!K}}}} + \frac{1}{{{J_{\!L}}}} = \frac{1}{{{J_{\!K}}}} + \frac{1}{{B{\omega ^{\frac{1}{2}}}}},\end{equation*}

(2) \begin{equation*}B = 0.62nF\!D_{{\rm O_2}}^{2/3}{\nu ^{ - 1/6}}{C_0},\end{equation*}

where *J*, *J*_L_ and *J*_K_ are the apparent current density, diffusion limiting current density and the kinetic-limiting current density, respectively; *ω* is the rotation rate of the disk electrode; *F* is the Faraday constant (96 485 C mol^−1^); *n* is the number of electrons transferred per O_2_ molecule; *C*_0_ is the bulk oxygen concentration in 0.1 M KOH solution (1.2 × 10^−6^ mol cm^−3^), ${D_{{\rm O_2}}}$ is the oxygen diffusion coefficient in 0.1 M KOH solution (1.9 × 10^−5^ cm^2^ s^−1^), *ν* is the kinematic viscosity of the 0.1 M KOH solution (0.01 cm^2^ s^−1^). With the constant including, *B* was calculated to be 0.11*n* (mA cm^−2^ s^1/2^). The calculations of *J*_K_ were based on the apparent current density (*J*) and electron transfer number (*n*) at 0.8 V (*vs.* RHE) with a rotation rate of 1600 rpm.

The methods applied to RRDE measurements were the same as those for the RDE measurements. The Pt-ring electrode potential was fixed at 0.5 V (*vs.* Ag/AgCl). The collection efficiency (*N*), which was measured under a nitrogen atmosphere using 0.1 M K_3_[Fe(CN_6_)] + 0.1 M KCl, was observed to be 0.397. The hydrogen peroxide yield (H_2_O_2_%) and *n* could be determined by the following equations:

(3) \begin{equation*}{\rm H_2}{\rm O_2}\left( {\rm{\% }} \right) = 200 \times \frac{{\frac{{{I_{\rm r}}}}{N}}}{{{I_{\rm d}} + \frac{{{I_{\rm r}}}}{N}}},\end{equation*}

(4) \begin{equation*}n = 4 \times \frac{{{I_{\rm r}}}}{{{I_{\rm d}} + \frac{{{I_{\rm r}}}}{N}}},\end{equation*}

where *I*_r_ is the ring current and *I*_d_ is the disk current.

The Tafel slope was analysed by the Tafel equation:

(5) \begin{equation*}\eta = {\rm a + b} \times {\rm log}\left| i \right|,\end{equation*}

(6) \begin{equation*}{\rm a} = \frac{{RT}}{{\alpha F}}{\rm log}{i^0},\end{equation*}

(7) \begin{equation*}{\rm b} = - \frac{{RT}}{{\alpha F}},\end{equation*}

where *η* is the overpotential, *i*^0^ is the exchange current density, *R* is the universal gas constant, *T* is the temperature, *α* is the transfer coefficient, and *F* is the Faraday constant.

### Measurement for the OER

The catalyst was first treated in a N_2_-saturated 0.1 M KOH solution by CV scans at 100 mV s^−1^ ∼10–20 cycles before the test. Then the OER current was tested by LSV scan in O_2_-saturated 0.1 M KOH with a sweep rate of 5 mV s^−1^ from 0.1 V to 0.9 V (*vs.* Ag/AgCl) after iR-compensation. The rotation rate was 1600 rpm. The OER electrocatalytic stability was tested by chronopotentiometry in the RDE apparatus at a constant current density of 5 mA cm^−2^ with the same rotation rate.

### Measurement of the EIS

The EIS was performed at an open circuit voltage, and the disturbance voltage was set to 5 mV in a frequency range from 100 kHz to 10 mHz.

### Zn–air battery performance tests

To evaluate the performance of Zn–air batteries, a home-made battery was constructed. Zn plate and carbon paper (3 cm $\times$ 3 cm) with gas diffusion film loaded catalyst ∼1 mg cm^−2^ were selected as anode and cathode, respectively. A mixed electrolyte solution consist of 10 mL 6 M KOH/0.2 M zinc acetate was filled between anode and cathode.

## Supplementary Material

nwz166_Supplemental_File
