# Supplementary material for: Encapsulating metal organic framework into hollow mesoporous carbon sphere as efficient oxygen bifunctional electrocatalyst
Source: Natl Sci Rev. 2019 Nov 11;7(3):609–19. doi: 10.1093/nsr/nwz166 (PMC8288918; doi:10.1093/nsr/nwz166)
Supplement: nwz166_Supplemental_File [file nwz166_supplemental_file.docx]

Supporting Information

Encapsulating Metal Organic Framework into Hollow Mesoporous Carbon Sphere as Efficient Oxygen Bifunctional Electrocatalyst

*Wanfeng Xiong^1,2^, Hongfang Li^1,*^ Hanhui You^1^, Minna Cao^1^, and Rong Cao^1,*^*

*1 State Key Laboratory of Structural Chemistry, Fujian Institute of Research on the Structure of Matter, Chinese Academy of Sciences, Fuzhou 350002, China.*

*2 College of Chemistry and Materials Science, Fujian Normal University, Fuzhou 350007, China*

** Corresponding authors. E-mail:* *rcao@fjirsm.ac.cn, hongfangli@fjirsm.ac.cn*

**Supplementary Figures**


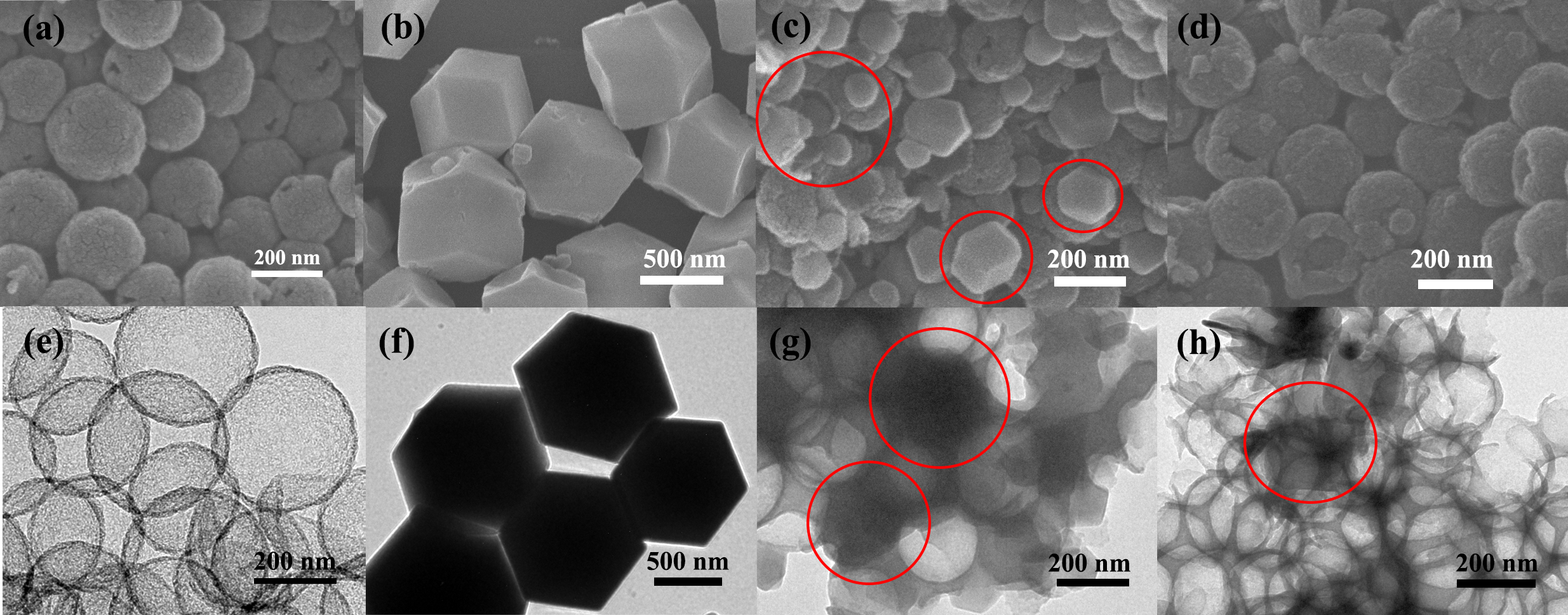


**Figure S1** SEM (top) and TEM (bottom) of (a, e) HMCS; (b, f) ZIF-67; (c, g) ZIF@HMCS-10%; (d, h) ZIF@HMCS-50%


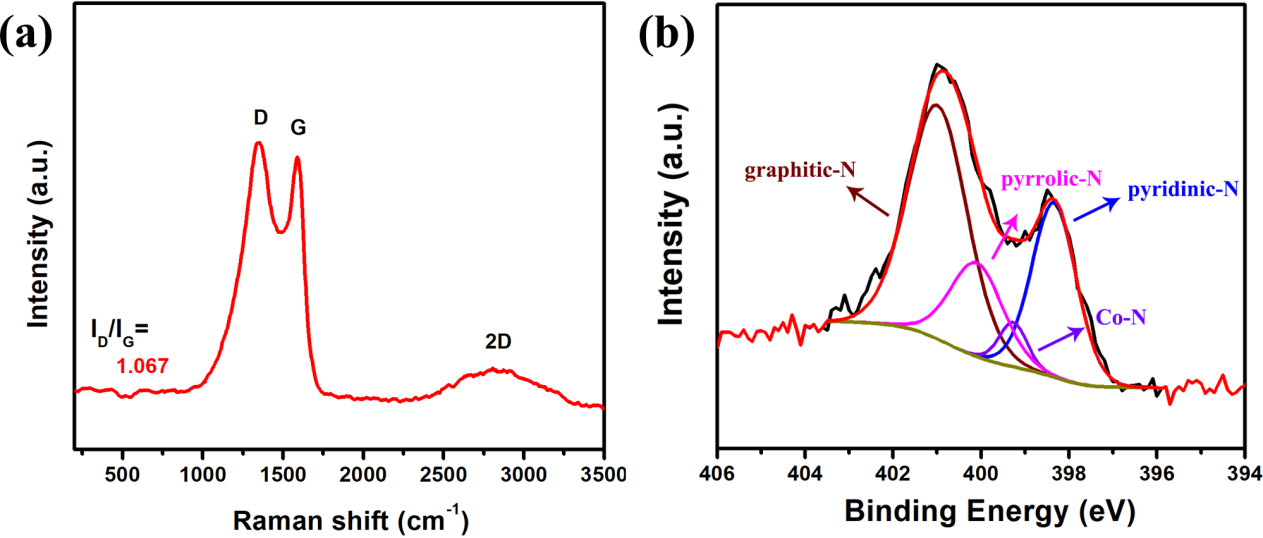


**Figure S2** (a) Raman spectrum of HMCS, (b) Deconvoluted XPS N 1s spectra of Co-HMCS.


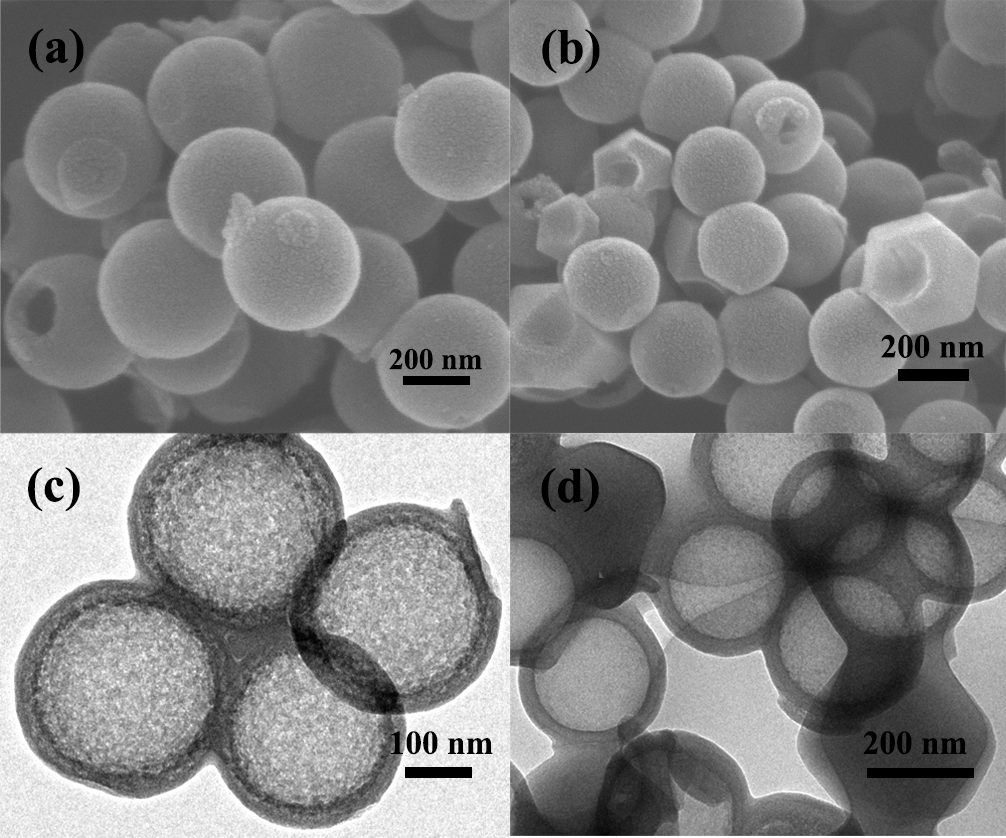


**Figure S3** SEM (top) and TEM (bottom) of (a, c) HMCS-1 and (b, d) ZIF@HMCS-1-25%


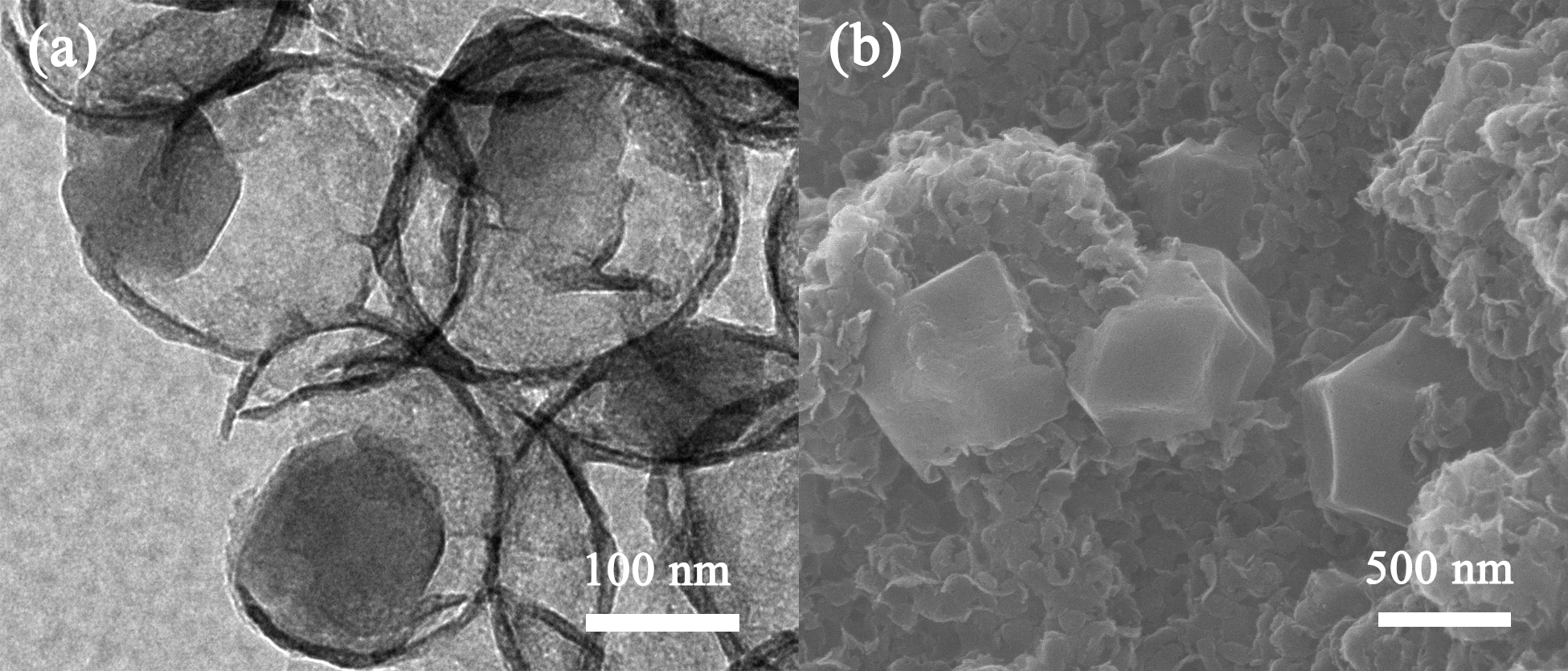


**Figure S4** (a) TEM of ZIF@HMCS-25% (150 ^o^C), (b) SEM of ZIF@BHMCS-25%


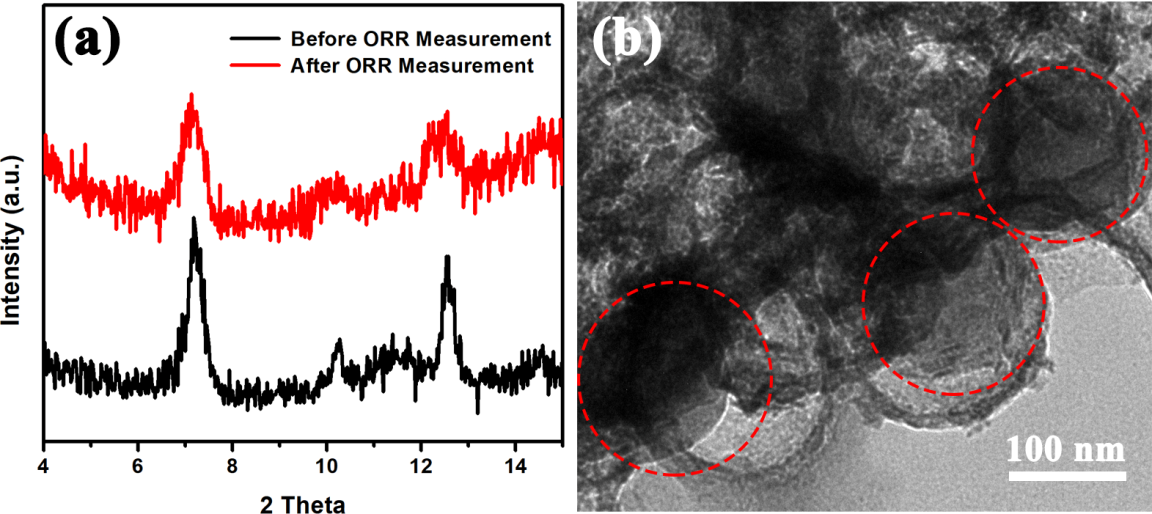


**Figure S5** (a) PXRD spectra before and after the ORR durability tests，(b) TEM image of ZIF@HMCS-25% after the ORR durability tests.


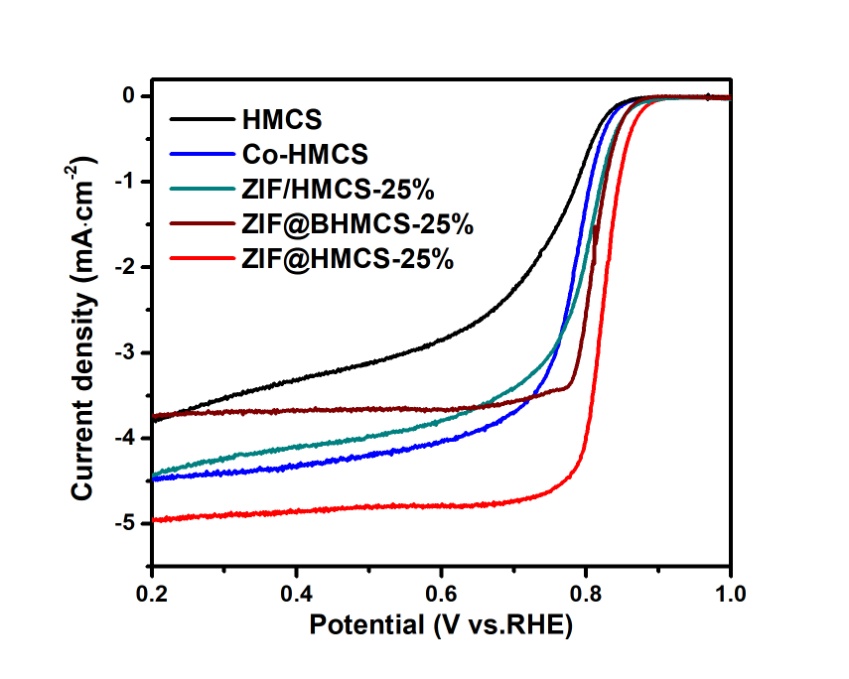


**Figure S6** LSV curves towars ORR for different samples .


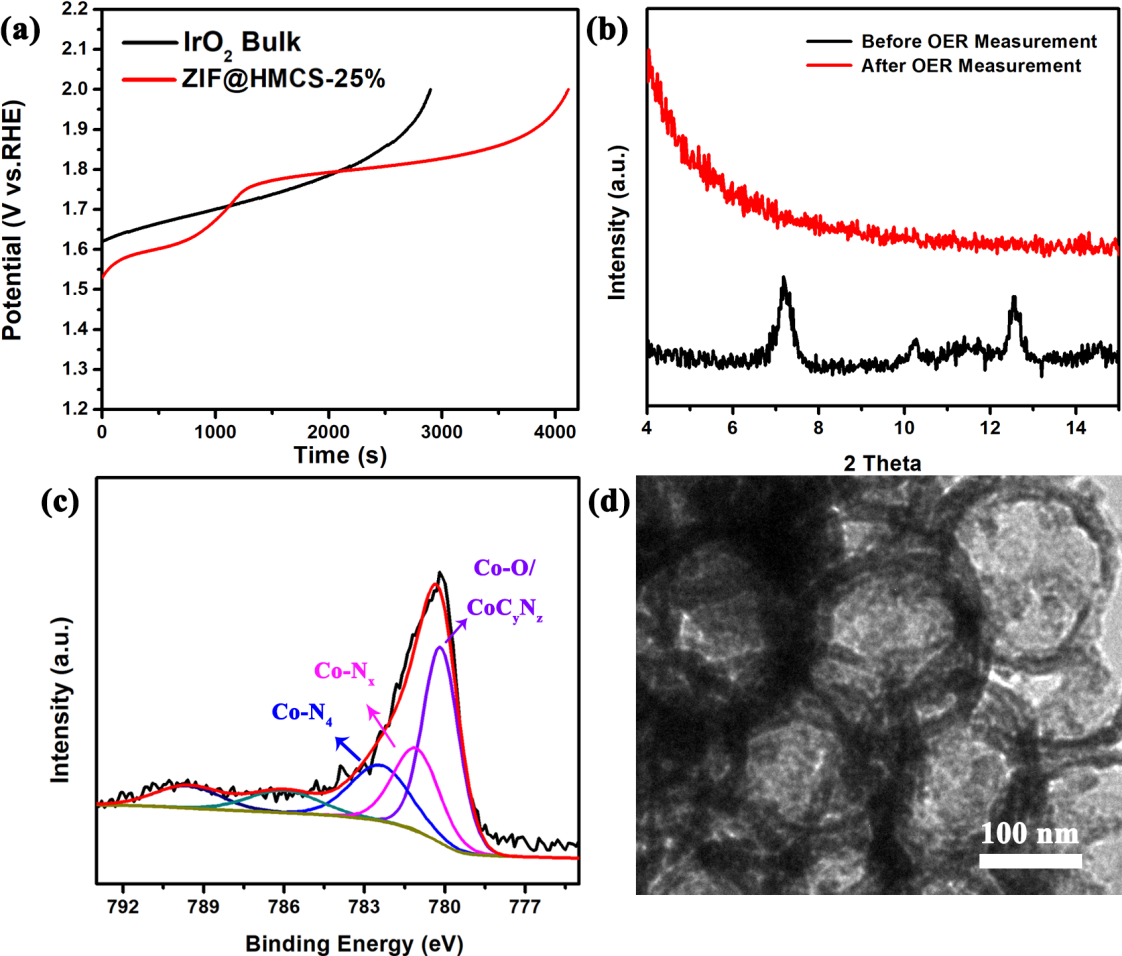


**Figure** **S7** (a) Chronopotentiometry responses of ZIF@HMCS-25% and IrO_2_ Bulk at 5 mA·cm^-2^, (b) PXRD spectra of ZIF@HMCS-25% before and after the OER chronopotentiometry responses, (c) XPS Co 2p_3/2_ spectra and (d) TEM of ZIF@HMCS-25% after the OER chronopotentiometry responses.

**
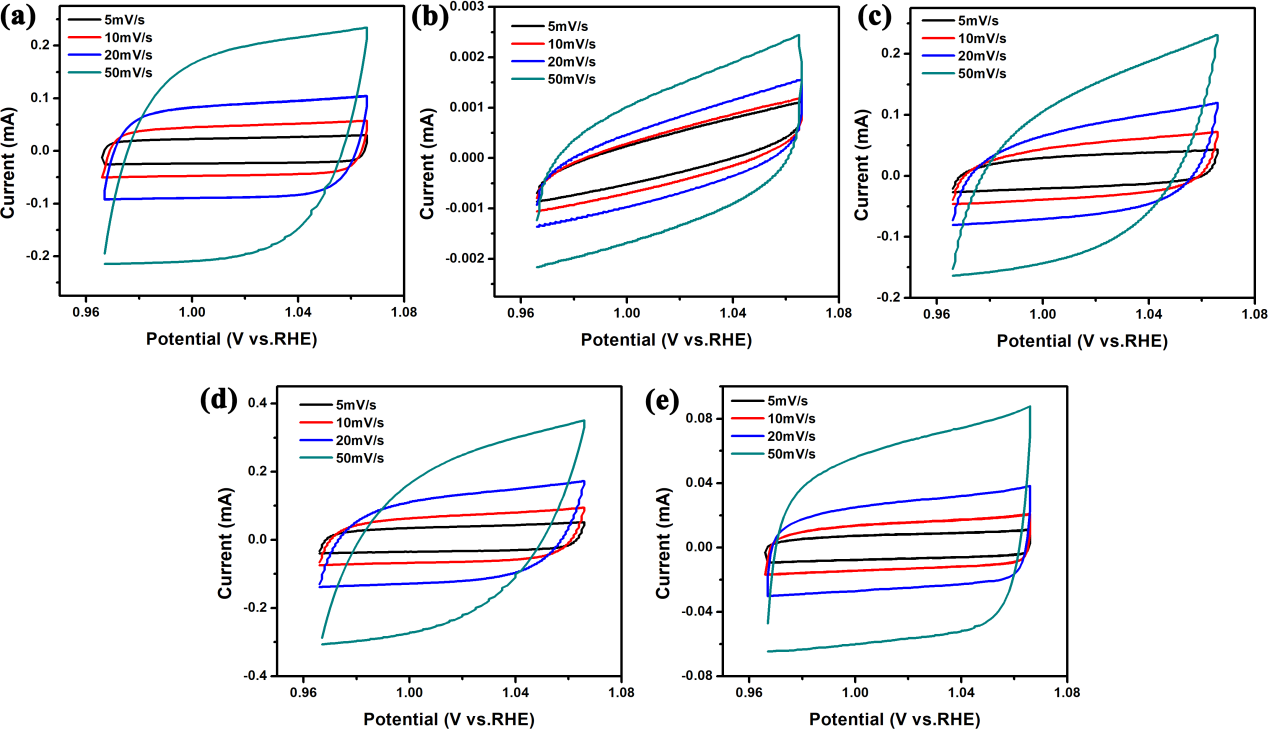
**

**Figure S8** CV curves measured at various sweep rates from 5 to 50 mV·s^-1^ (a) HMCS; (b) ZIF-67; (c) ZIF@HMCS-10%; (d) ZIF@HMCS-25%; (e) ZIF@HMCS-50%.

**
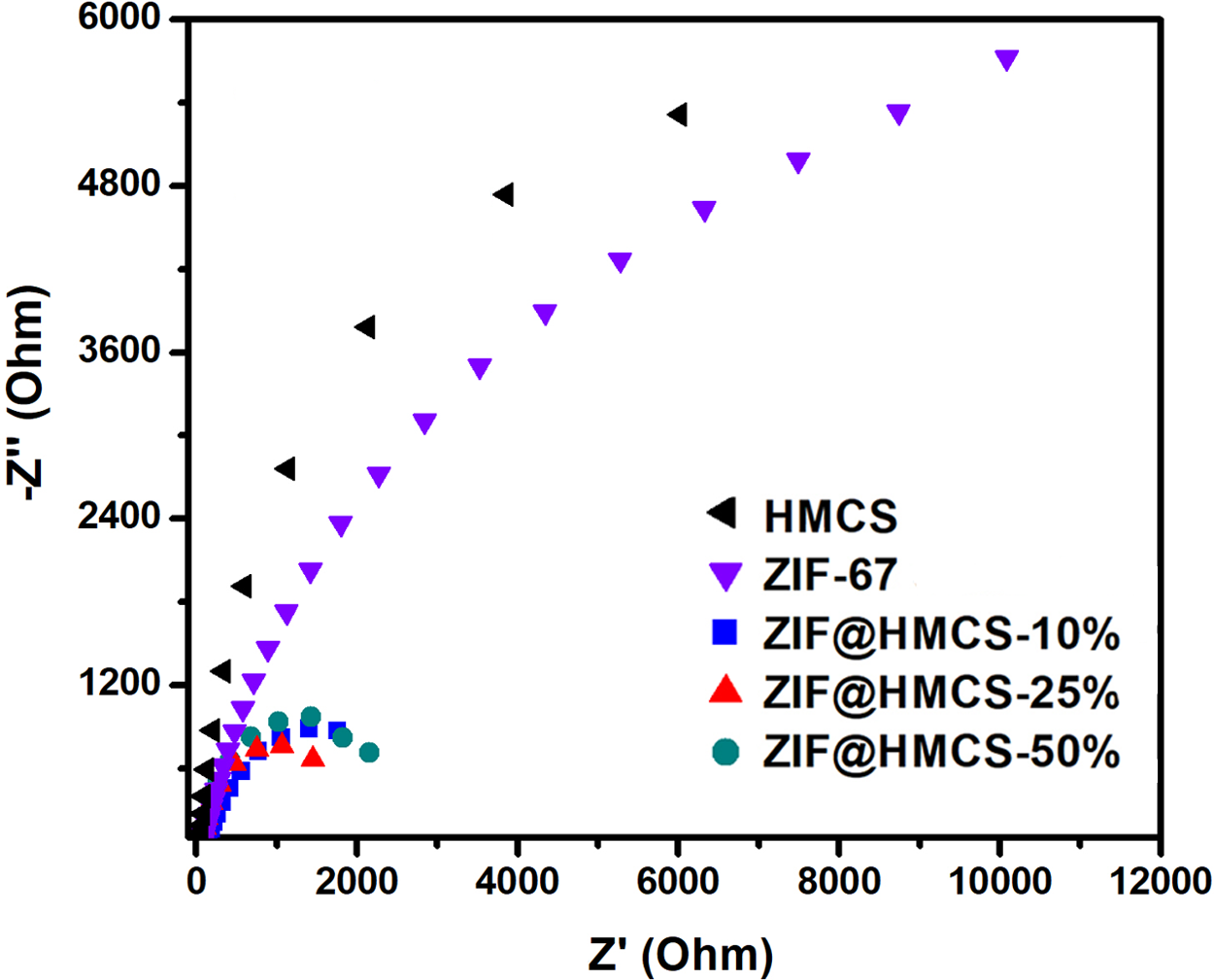
**

**Figure S9** Electrochemical impedance spectra (EIS) low frequency range of various samples

**Supplementary Tables**

**Table S1.** The physical characterization of the samples

| Samples | Co content  [%]^a)^ | N content  [%]^b)^ | S_BET_  [m^2^ g^-1^] | Micropore Volume  [cm^3^ g^-1^]^c)^ | Mesopore Volume  [cm^3^ g^-1^]^d)^ |
| --- | --- | --- | --- | --- | --- |
| HMCS | NA | 6.4 | 916 | 0.04 | 2.03 |
| ZIF-67 | 23.0 | 20.8 | 1295 | 0.67 | 0.01 |
| ZIF@HMCS-10% | 14.2 | 18.7 | 1017 | 0.39 | 0.67 |
| ZIF@HMCS-25% | 9.3 | 14.2 | 950 | 0.33 | 1.06 |
| ZIF@HMCS-50% | 6.3 | 10.7 | 820 | 0.21 | 1.29 |

^a)^ Co content was measured by ICP;

^b)^ N content was measured by elemental analysis;

^c)^ micropore volume was obtained by t-Plot micropores;

^d)^ mesopore volume was obtained by BJH.

**Table S2.** Comparisons of different catalysts’ catalytic activities[1-7]

| **Electrocatalysts** | **ORR *E_1/2_***  **(V *vs* RHE)** | **ORR *J_L_* _at 0.8V_**  **mA/cm^2^** | **ORR Tafel slope**  **(mV·dec^-1^)** | **OERη_at 10 mA·cm_^-2^**  **(mV)** | **OER Tafel *slop*e**  **(mV·dec^-1^)** | **Ref.** |
| --- | --- | --- | --- | --- | --- | --- |
| ZIF-67 | 0.681 | -3.22 | 70.3 | - | 172.2 | This work |
| HMCS | 0.684 | -3.81 | 49 | - | 385.8 | This work |
| ZIF@HMCS-10% | 0.777 | -4.03 | 45.7 | 419 | 163.7 | This work |
| ZIF@HMCS-25% | 0.823 | -4.98 | 41.3 | 407 | 99.3 | This work |
| ZIF@HMCS-50% | 0.783 | -4.55 | 49.8 | 447 | 146.6 | This work |
| 20% Pt/C | 0.832 | -5.3 | 72.5 | - | - | This work |
| IrO_2_ Bulk | - | - | - | 420 | 80.2 | This work |
| Co-HMCS | 0.779 | -4.36 | - | - | - | This work |
| ZIF/HMCS-25% | 0.793 | -4.28 | - | - | - | This work |
| ZIF@BHMCS-25% | 0.803 | -3.68 | - | - | - | This work |
| Co^II^_3_Co^III^_2_(im)_12_ | - | - | - | 496 | 82 | Chemistry–A European Journal, 2016, **22**, 3676-3680. |
| Ti_3_C_2_T_x_/Co BDC |  | - | - | 410 | 48.2 | *ACS Nano*, 2017, **11**, 5800-5807 |
| ε-MnO_2_/MIL-100(Fe) | 0.64 | -5.56 | 117 | - | - | *Journal of Materials Chemistry A*, 2015, **3**, 16168-16176 |
| (G-dye-Fe P)_n_MOF | 0.78 | -6.3 | - | - | - | *J Am Chem Soc*, 2012, **134**, 6707-6713 |
| Co-MOF@CNTs | 0.82 | -4.2 | - | 347 | 69 | *Journal of Power Sources*, 2016, **326**, 50-59 |
| C-MOF-C2-900 | 0.817 | -6.1 | - | 350 | 79 | *Adv Mater*, 2018, **30** |
| Co@NC | 0.79 | -4.15 | - | 480 | 99 | *Journal of Materials Chemistry A*, 2016, **4**, 15836-15840. |

**Table S3.** Impedance information obtained by fitting from 100 KHz to 100 Hz with the equivalent circuit

| **Samples** | ***R_s_* (Ohm)** | **rel. std. error (%)** | ***R_ct_*(Ohm)** | **rel. std. error (%)** |
| --- | --- | --- | --- | --- |
| HMCS | 41.03 | 0.164 | 2.47 | 3.319 |
| ZIF-67 | 49.5 | 0.924 | 33.87 | 24.6 |
| ZIF@HMCS-10% | 43.9 | 0.185 | 9.01 | 16.5 |
| ZIF@HMCS-25% | 44.3 | 0.105 | 3.58 | 5.72 |
| ZIF@HMCS-50% | 45.01 | 0.15 | 2.93 | 3.86 |

**References:**

1. Fang, Y, Li, X, Li, F*, et al.* Self-assembly of cobalt-centered metal organic framework and multiwalled carbon nanotubes hybrids as a highly active and corrosion-resistant bifunctional oxygen catalyst. *Journal of Power Sources*. 2016; **326**: 50-9.

2. Flugel, EA, Lau, VW, Schlomberg, H*, et al.* Homonuclear Mixed-Valent Cobalt Imidazolate Framework for Oxygen-Evolution Electrocatalysis. *Chemistry–A European Journal*. 2016; **22**(11): 3676-80.

3. Jahan, M, Bao, Q, Loh, KP. Electrocatalytically active graphene-porphyrin MOF composite for oxygen reduction reaction. *J Am Chem Soc*. 2012; **134**(15): 6707-13.

4. Li, X, Jiang, Q, Dou, S*, et al.* ZIF-67-derived Co-NC@CoP-NC nanopolyhedra as an efficient bifunctional oxygen electrocatalyst. *Journal of Materials Chemistry A*. 2016; **4**(41): 15836-40.

5. Wang, H, Yin, F, Chen, B*, et al.* Synthesis of an ε-MnO_2_/metal–organic-framework composite and its electrocatalysis towards oxygen reduction reaction in an alkaline electrolyte. *Journal of Materials Chemistry A*. 2015; **3**(31): 16168-76.

6. Zhang, M, Dai, Q, Zheng, H*, et al.* Novel MOF-Derived Co@N-C Bifunctional Catalysts for Highly Efficient Zn-Air Batteries and Water Splitting. *Adv Mater*. 2018; **30**(10): 1705431.

7. Zhao, L, Dong, B, Li, S*, et al.* Interdiffusion Reaction-Assisted Hybridization of Two-Dimensional Metal-Organic Frameworks and Ti_3_C_2_T_x_ Nanosheets for Electrocatalytic Oxygen Evolution. *ACS Nano*. 2017; **11**(6): 5800-7.
